# Normalization Strategies in Multi-Center Radiomics Abdominal MRI: Systematic Review and Meta-Analyses

**DOI:** 10.1109/OJEMB.2023.3271455

**Published:** 2023-04-28

**Authors:** Jovana Panic, Arianna Defeudis, Gabriella Balestra, Valentina Giannini, Samanta Rosati

**Affiliations:** Department of Surgical Science, and Polytechnic of Turin, Department of Electronics and TelecommunicationsUniversity of Turin 9314 10129 Turin Italy; Department of Surgical ScienceUniversity of Turin 9314 10129 Turin Italy; Candiolo Cancer InstituteFPO-IRCCS 18524 10060 Candiolo Italy; Department of Electronics and TelecommunicationsPolytechnic of Turin 19032 10129 Turin Italy

**Keywords:** Abdominal MRI, artificial intelligence, multi-center database, normalization, radiomics

## Abstract

*Goal:* Artificial intelligence applied to medical image analysis has been extensively used to develop non-invasive diagnostic and prognostic signatures. However, these imaging biomarkers should be largely validated on multi-center datasets to prove their robustness before they can be introduced into clinical practice. The main challenge is represented by the great and unavoidable image variability which is usually addressed using different pre-processing techniques including spatial, intensity and feature normalization. The purpose of this study is to systematically summarize normalization methods and to evaluate their correlation with the radiomics model performances through meta-analyses. This review is carried out according to the PRISMA statement: 4777 papers were collected, but only 74 were included. Two meta-analyses were carried out according to two clinical aims: characterization and prediction of response. Findings of this review demonstrated that there are some commonly used normalization approaches, but not a commonly agreed pipeline that can allow to improve performance and to bridge the gap between bench and bedside.

## I. Introduction

Currently, the role of medical imaging is evolving from being mainly a diagnostic tool to gaining a central role in the context of personalized precision medicine [1]. This paradigmatic shift was made possible by the development of radiomics, which allows the extraction, from medical images, of quantitative features providing useful information on diagnosis and prognosis [1], [2]. Despite the encouraging results provided by recent studies [3], [5], [6], there is still no radiomics-based systems used in clinical practice for abdominal imaging. This is mainly due to the lack of multi-center clinical trials for both system development and validation [7]. The acquisition of images from several institutions is surely complex for many technical reasons, in addition to legal, ethical and administrative issues [8]. From the technical point of view, the most relevant obstacle is represented by the unavoidable high image intensity distribution variability due to different scanners, acquisition protocols, reconstruction settings and the patients’ characteristics. This issue is particularly relevant when a multi-center dataset is used, and multi-center validation is compelling for developing robust, reproducible, and statistically relevant results.

Different standardization guidelines have been proposed to address the above-mentioned problem in the case of computer tomography (CT) and positron emission tomography (PET)/CT imaging, while they are not available for Magnetic Resonance Imaging (MRI) [9]. However, signal intensities of MRI are non-standardized and highly dependent on manufacturer and acquisition protocol parameters [10]. Therefore, bigger efforts are needed to solve problems related to low repeatability and reproducibility.

Currently, different pre-processing algorithms have been implemented to reduce the variability [11], [12], [13], especially in studies involving retrospective databases when it is not feasible to use standardized imaging acquisition protocols [1]. The most used approach is normalization, including a set of techniques in which values are shifted and/or rescaled, and that could be applied to parameters related to different image characteristics, i.e., physical dimensions (spatial normalization), pixels intensity (intensity normalization) and radiomics features (feature normalization).

The aim of this study is to systematically review normalization approaches applied to multi-center abdominal MRI, to assess whether it is possible to provide guidelines or evidences about the performances of the most frequently used methods. Even if this topic was widely addressed for the brain [14], [15], to the best of our knowledge, there is no systematic review regarding the abdominal area in the literature. As a secondary endpoint, we conducted different meta-analyses to understand the impact of normalization methods on radiomics models, according to their aim.

## II. Methods

### A. Search Strategy

This review was carried out according to the Preferred Reporting Items for Systematic Reviews and Meta-Analyses (PRISMA) statement. Relevant articles were identified by searching three databases: PubMed, Web of Science and Scopus. The query used for the literature search was “MRI” AND “multicenter” AND (“database” OR “trial” OR “standardization” OR “normalization”) AND (“radiomics” OR “Artificial Intelligence” OR “Machine Learning” OR “Deep Learning”).

Literature searching, study identification, and data extraction from eligible studies were performed by one investigator with experience in abdominal radiomics field research (J.P.).

### B. Eligibility Criteria

Searched studies had to further come across the following eligibility criteria to be incorporated in the present review: (i) written in English; (ii) AI radiomics-based studies; (iii) published between January 2012 (when the radiomics definition has been first published by Lambin et al. [2]) and December 2022; (iv) based on a multi-center MRI database; (v) original research work published in a peer-reviewed journal.

### C. Exclusion Criteria

Studies were excluded based on any of the following criteria: (i) papers assessing image quality, (ii) papers describing only harmonization or standardization acquisition pipelines without using them for the development of a radiomics signature or AI model for detection/characterization/prognosis, (iii) papers describing challenges or online databases, and (iv) not clearly specifying the pre-processing step.

### D. Papers Analysis

For all included papers, the following information was collected (when available either on the manuscript or in supplementary materials): organ of interest, clinical aim, publication year, database details, normalization approach and method, radiomics features extracted, developed radiomics model, distinguishing between Machine and Deep Learning (ML and DL), and performances on the validation set. We grouped clinical aims in three categories: (i) detection as the capability to detect the pathologic tissue, i.e., its presence, and, if confirmed, its localization on the medical image; (ii) characterization as the ability to predict the clinical outcome; and (iii) prediction of therapy response, only appliable on pathologic tissues.

### E. Meta-Analysis

We conducted different meta-analyses on the model performances, according to the aim, i.e., detection, characterization, response to therapy. We considered the area under the curve (AUC) and its Confidence Interval (CI) as the performance metric, which was the most frequently evaluated parameter among the studies. We excluded papers that did not report AUC and neither its standard deviation, standard error or CI values, and that weren't validated on an external dataset, since this does not determine model reproducibility and generalizability to new and different patients [16].

The random-effects model was used to calculate the pooled AUC and to produce the forest plot. Cochran's Q and the I^2^ statistic were calculated. Cochran's Q statistic tests the studies' heterogeneity, under the null hypothesis H_0_ that all studies are homogeneous (a *p-value* < 0.05 was considered statistically significant). The I^2^ value was used to quantify heterogeneity, providing an estimate of the percentage of variability among included studies: values of 25% and less are usually considered to be low, 25%–50% moderate and above 75% are considered high. In case of detected heterogeneity, a moderator analysis was carried out by dividing studies into subgroups according to the organ, for each aim. Subgroups containing only one paper were excluded from the meta-analysis. The weight of each study was calculated with the inverse variance method, in which the weight given to each study is inverse of the variance of the effect estimate, minimizing the uncertainty of the pooled effect estimate [17]. The meta-analysis and the described statistical analyses were performed using R and the *metafor* package [18].

## III. Results and Discussions

### A. Papers Collection

**Fig. 1.**
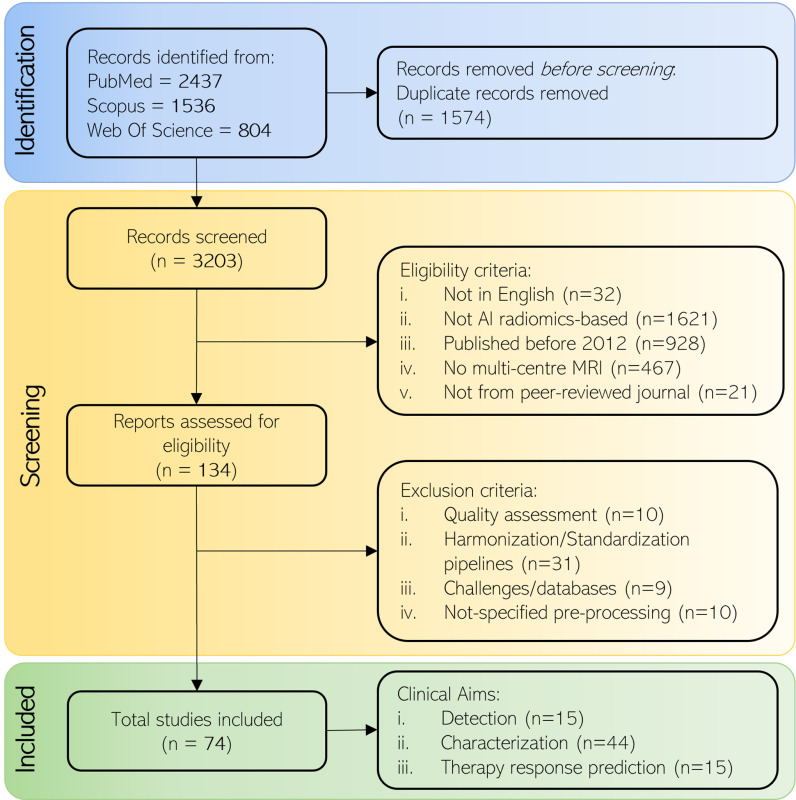
Flow chart of the selection process of the studies included in the present review. At first, the author searched the 3 databases to identify the relevant articles for the study (Identification). Then, she screened the initially obtained studies considering the eligibility and exclusion criteria (Screening), and finally, selected the articles used for the study, based on the research objectives (Included).

During the identification step, 4777 papers were collected from three search databases. Among them 1574 were duplicate records, therefore they were excluded resulting in a total of 3203 papers. During the screening step, 3069 were excluded as they did not meet the eligibility criteria. Of the remaining 134 papers, 60 were eliminated after reviewing the study context and design. Finally, 74 papers were included in the final analysis. Fig. 1 reports the selection process and the number of papers per aim. All comparison tables reporting the normalization approaches, features, models, and performances are included in the supplementary materials, one for each organ.

### B. Clinical Analysis

**Fig. 2.**
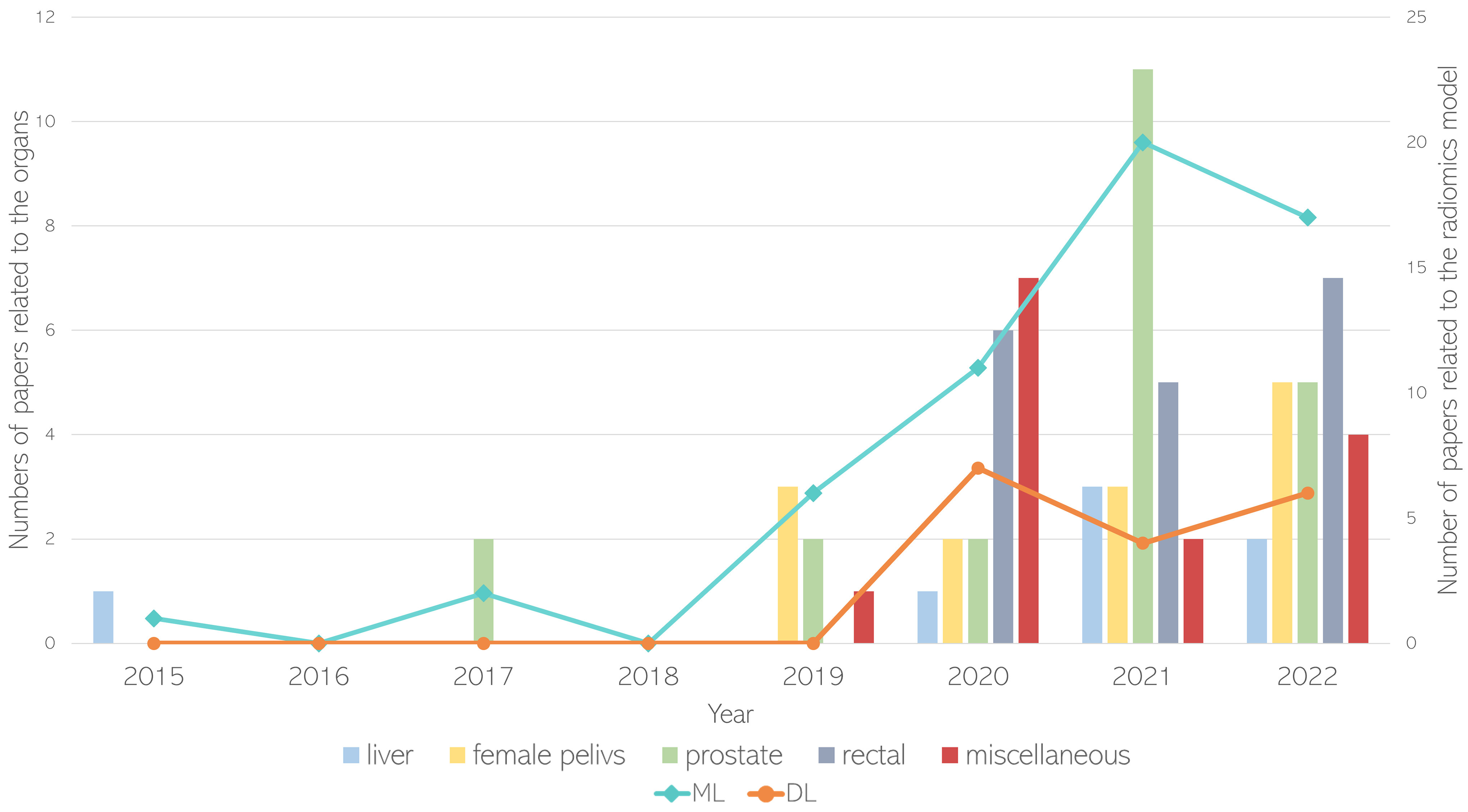
This combined graph shows the distribution of the number of papers related to the abdominal organs (bars) and radiomics models (lines) from 2015 to 2022.

All included papers have been published since 2015, with an increase from 2019. The most studied organs are the prostate [3], [19], [20], [21], [22], [23], [24], [25], [26], [27], [28], [29], [30], [31], [32], [33], [34], [35], [36], [37], [38], [39], [40] (S-Table I), female pelvis [4], [41], [42], [43], [44], [45], [46], [47], [48], [49], [50], [51], [52] (S-Table II), and rectum [5], [53], [54], [55], [56], [57], [58], [59], [60], [61], [62], [63], [64], [65], [66], [67], [68], [69] (S-Table III), while a lower number of publications were related to the liver [6], [70], [71], [72], [73], [74], [75] (S-Table IV), and a miscellaneous group of organs including kidney [76], [77], [78], [79], [80], bladder [81], [82], pancreas [83], [84], and soft abdominal tissues [85], [86], [87], [88], [89] (S-Table V). As shown in Fig. 2, the first study applying ML on multi-center databases was published in 2015 for the characterization of liver fibrosis, classifying them into five groups, ranging from no-fibrosis (0) to cirrhosis (5) [70]. Subsequently, two studies were published in 2017 developing ML models for prostate cancer detection: one assessing the differences between the transactional and peripherical zone of the prostate [40], while the other providing useful information for radiotherapy dose differentiation treatments [36].

The turning year, in which the number of papers showed a big rise also in other abdominal organs, was 2020. This might be due to the increasing number of public databases [90], [91], [92] and/or collaboration between institutions. Moreover, also a growing interest related to DL models was observed in the literature from this year. Considering the clinical question, as shown in the supplementary materials, ML is used to non-invasively characterize pathological tissues in 67% of the papers (38/57), predict the therapy response in 25% (14/57) and detect the disease in 8% (5/57), while DL is mostly used for detection (10/17 of papers) while only 6/17 (35%) and 1/17 (6%) of papers focus on characterization and therapy response, respectively.

### C. Technical Analysis

**Fig. 3.**
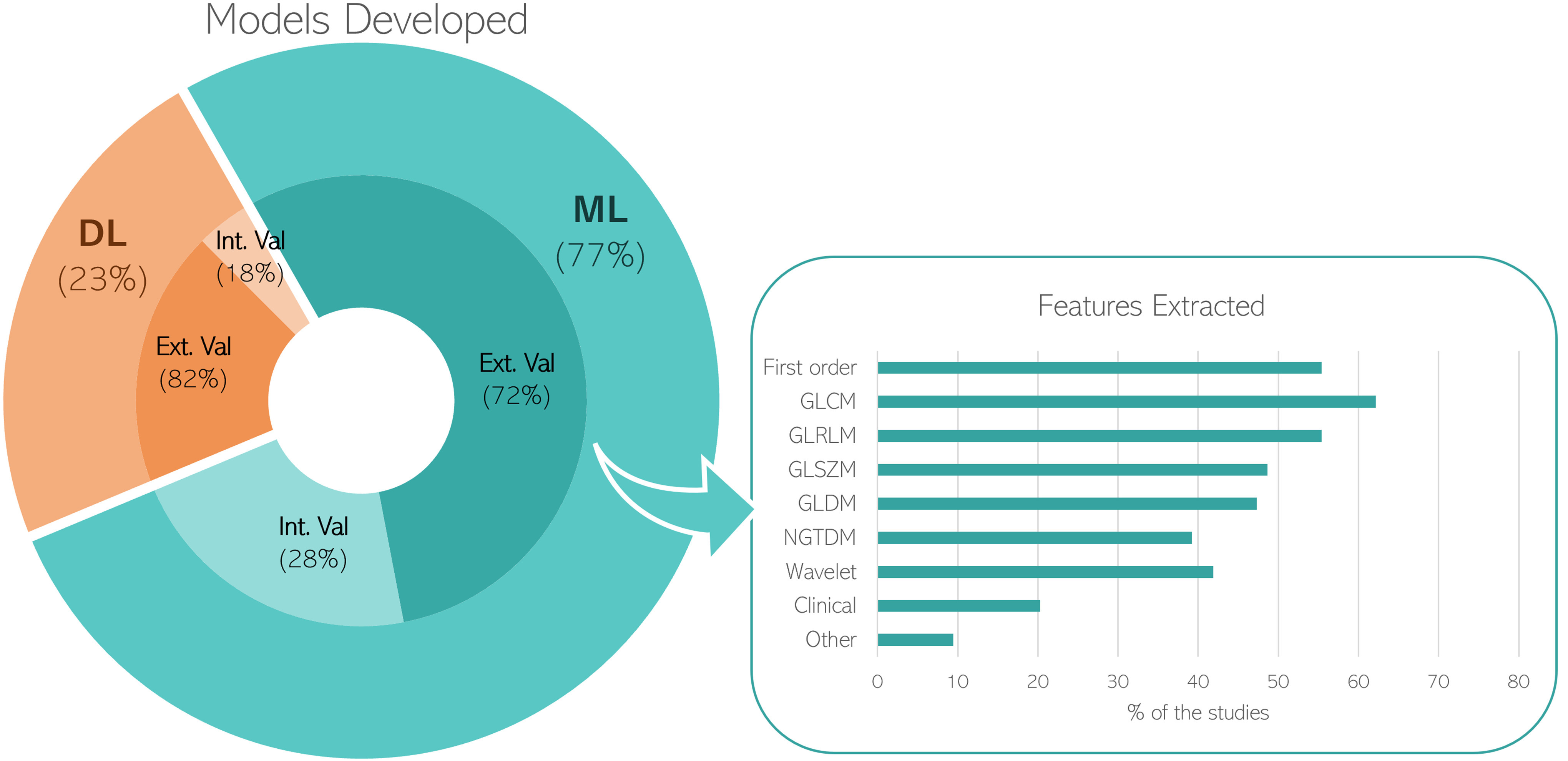
The pie graph (on the left) shows the distributions of the models developed among the included papers. Specifically, the percentage related to the internal (Int.) and external (Ext.) validations are presented for both Machine Learning (ML) (blue) and Deep Learning (DL) (orange). The bar diagram (on the right) shows the percentage of different radiomics feature groups extracted in the ML studies.

Fig. 3 shows that 77% of papers developed ML radiomics systems, while the remaining 23% were based on DL. In both cases, most of the studies (>70%) used the multi-center database to externally validate the model. Focusing on the ML systems, we analyzed the extracted radiomics feature groups. As shown by the bar diagram, the Gray-Level Co-Occurrence Matrix (GLCM) is the most used one, followed by the Gray-Level Run Length Matrix (GLRLM) and the First Order. Interestingly, since 2021 deep learning features [93] were introduced, e.g., Hiremath et al. [22] and Liu et al. [94].

Of note, since 2019, 15/74 papers include clinical features, available from routine practice, into their model in order to increase the predictive potential usefulness (S-Table I–V).

#### 1) Normalization Approaches

The following normalization approaches were used on MRI datasets:

• *Spatial normalization*: a process that changes the spatial characteristics of the image, e.g., the pixel's resolution, the Field of View (FOV), sequences orientation, etc. All methods apply geometrical transformations (e.g., resampling to obtain a fixed pixel resolution), which do not need normalization parameters. This approach allows obtaining images including almost the same anatomical regions, thus avoiding histogram mismatches due to different FOVs rather than signal intensity dissimilarities. However, spatial modifications may alter the organs' morphology, undermining the clinical significance.
• *Intensity normalization*: a process that rescales the pixel intensity values to the same range. Most methods allow evaluating the normalization parameters independently on the sequences, except those which need a normalization reference, e.g., *hist_norm* [95], and *NyulUdupa* [96]. At the same time, modifying the histogram distribution may alter the clinical significance of both healthy and pathological tissues.
• *Feature normalization*: a process that rescales values of the radiomics feature extracted to the same range. On one hand, all methods allow reducing the differences between the features, without altering the clinical significance. On the other hand, a training set is needed to evaluate the normalization parameters.

**Fig. 4.**
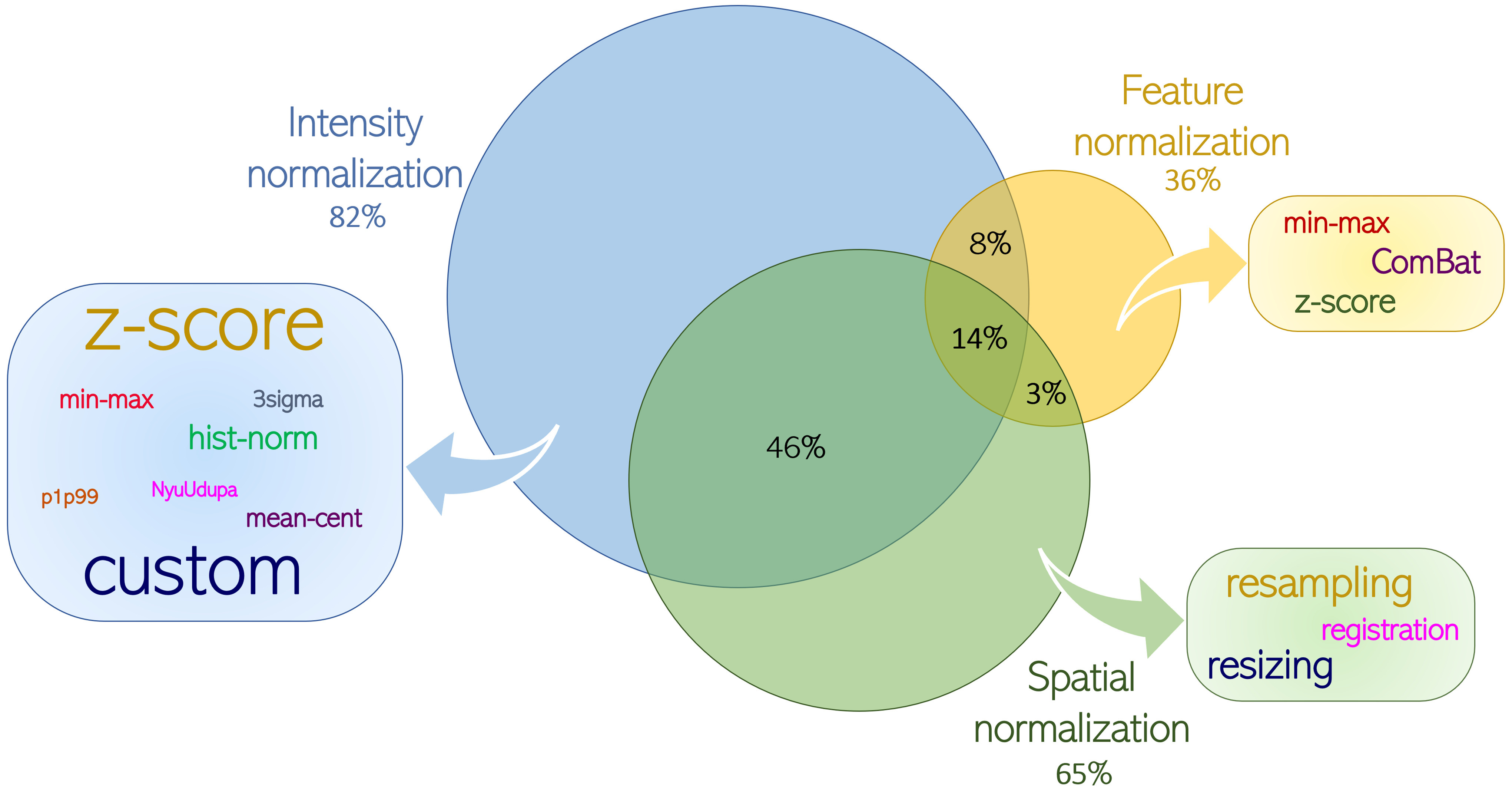
This is the knowledge map of the most used normalizations among the included papers, showing the main approaches and methods followed. Each node represents a normalization approaches, while the boxes include the methods adopted. For each node it is showed the rate of each approach separately, and the rate of multi-approach normalizations. All rates are evaluated with respect to the amount of the included papers. The sizes of the words denote the number of publications related to the method. The dark blue line groups the total amount of the included papers.

Among all, intensity normalization is applied on 61/74 (82%) papers, being the most frequent, while the feature and spatial normalizations on 27/74 (36%) and 48/74 (65%) papers, respectively. In 52/74 studies the images underwent more than one normalization approach: 6/74 (8%) both intensity and feature, 34/74 (46%) spatial and intensity, 2/74 (3%) spatial and feature, and 10/74 (14%) all three approaches. Colored boxes in Fig. 4 list the normalization methods used for each approach.

In general, three methods are currently used for spatial normalization: *registration* to align the sequences and reduce the motion artefacts, *resampling* to obtain the same resolution, and *resizing* to obtain the same size for all images. The most commonly applied method is *resampling* (31/48), which has been used individually in 12/16 studies on the prostate, 4/5 on the liver, and 6/9 in the miscellaneous group. Moreover, it has been applied in combination with at least another spatial normalization method in 14/48 papers, i.e., *resampling* and *resizing* on the female pelvis (4/9), and the rectum (3/9). *Registration* is the least frequently used method (10/48) among all organs. For intensity normalization, the two most used methods are the standardization (*z-score*) of the intensity distribution, which is applied on 21/61, and *custom* algorithms on 31/61, including normalization using pre-defined values, discretization of the distributions, the use of Advanced Normalization Tools (ANTs) or filters, the application of DL methods for harmonizing the image, or the combination of more than one method. The remaining 9/61 papers applied one of the following methods: *min-max* or *3sigma* scaling and mean centering (*mean-cent*), and histogram normalization (*hist-norm*) which matches the original distribution to a histogram reference (e.g., healthy subject, other organs, etc.). Most papers related to the prostate (10/20) and miscellaneous (8/10) have developed custom methods for the intensity normalization, while the *z-score* is frequently applied to female pelvis (5/10) and rectal (7/16), and *hist-norm* on the liver (2/5). More details are in S-Table I–V. Regarding feature normalization, only three methods have been found in the included papers and are almost equally used: i.e., rescaling according to the minimum and maximum feature values (*min-max*) is applied on 6/27 papers, standardization (*z-score*) on 9/27, and harmonization using *ComBat* [12] on 5/27. The first two methods are very easy to implement and could be applied on different centers without a training phase, i.e., using the same normalization parameters derived from the previously included dataset. Conversely, the *ComBat* method can only normalize features across different centers by using values computed on a subset of data from each center that will be normalized. Therefore, it requires available and labelled data from all centers/scanners included in the dataset, not allowing the direct application of previously determined normalization parameters on external validation cohorts [8]. Of note, 5/27 papers declared they applied a features normalization approach without specifying which method, and 1/27 combined both *ComBat* and *z-score*. Observing the S-Tables I–V, the *z-score* is mostly applied on the rectum (3/6), the *min-max* on the prostate (3/5), while the *ComBat* on the female pelvis (4/7).

### D. Meta-Analysis

The 22 papers included in the meta-analysis are reported in the supplementary tables in bold. We did not perform the meta-analysis on detection, since there were not enough studies that could be included for this purpose.

**Fig. 5.**
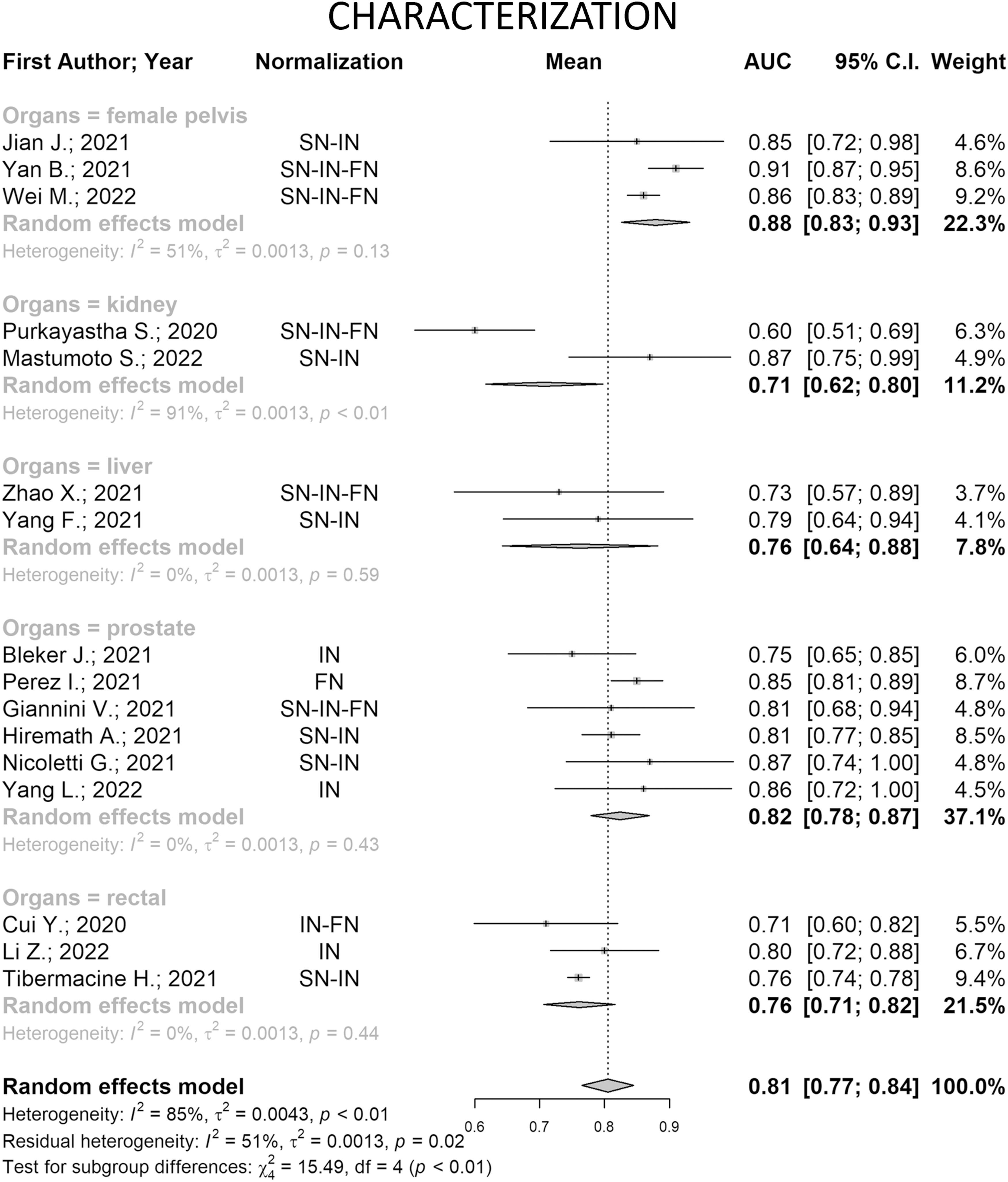
Forest plot of the studies addressing characterization aim for the pooled area under the curve (AUC) and 95% confidence interval (CI). Horizontal CI lines represent 95% confidence interval of the point estimates, while the vertical dash represents the overall pooled AUC. The diamond represents the pooled AUC and its 95% CI obtained for each subgroup and considering the 14 papers all together. Note: Intensity Normalization (IN), Spatial Normalization (SN), Feature Normalization (FN).

Concerning the 16 papers addressing the characterization (Fig. 5), the studies are statistically heterogeneous (heterogeneity = 85%, *p-value* < 0.01), and differences are partially explainable using the organs as the moderator (residual heterogeneity: 51%, *p-value* = 0.02).

Considering each organ separately, only the kidney showed high significant heterogeneity between the two included studies (*p-value* < 0.01), which differ in that one of them applies features normalization in addition to spatial and intensity normalizations. This behavior might suggest that in this case normalizing the features does not improve the performance (AUC = 0.60 vs AUC = 0.87), however, the sample size is very small to strongly assume this point. It is noteworthy that the papers related to the liver, rectal and prostate show very low heterogeneity (I^2^ = 0% for the three organs) even though they applied very different normalization approaches. This might suggest that there is no preferable normalization strategy when dealing with these organs. Considering average results, two organs reach an average AUC higher than the overall pooled AUC, i.e., female pelvis (0.88 vs 0.81) and prostate (0.82 vs 0.81). It is noteworthy that, in studies regarding the female pelvis papers, *ComBat* feature normalization seems to slightly increase performances (AUC = 0.86 and 0.91 vs 0.85). However, there is not a clear correlation between the used approach and performance, therefore it is not possible to define a pathway for MRI variability reduction.

**Fig. 6.**
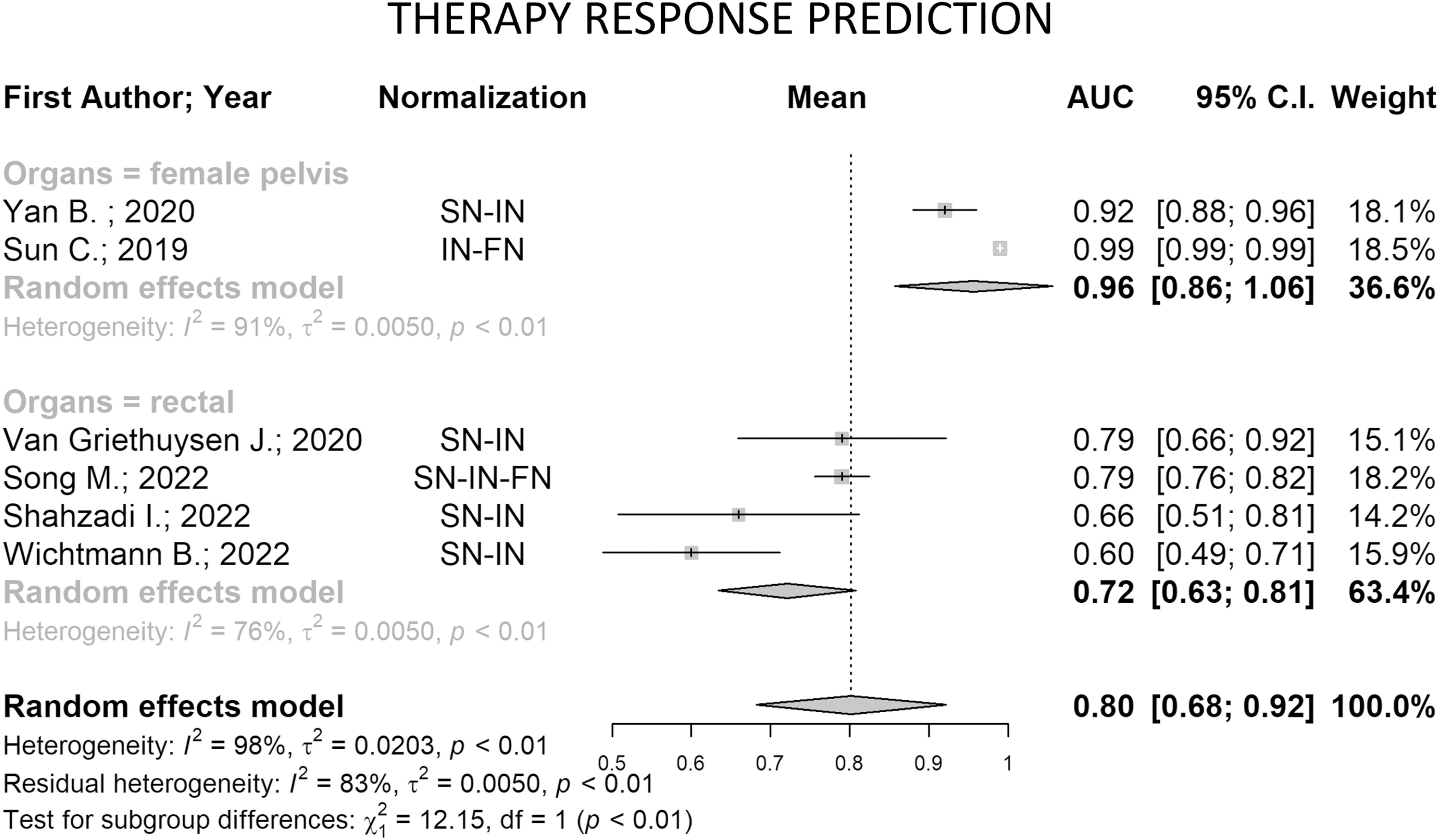
Forest plot of the studies addressing the therapy response prediction aim for the pooled area under the curve (AUC) and 95% CI. Horizontal lines represent 95% confidence interval of the point estimates, while the vertical dash represents AUC of individual studies. The diamond means the pooled AUC of each subgroup and of all 6 papers. Note: Intensity Normalization (IN), Spatial Normalization (SN), Feature Normalization (FN).

Concerning the 6 papers addressing the prediction of response to therapy (Fig. 6), the overall study heterogeneity is 98% (*p-value* < 0.01), partially explainable using the organs as the moderator (residual heterogeneity: 83%). In this case, the performances of the two groups were statistically different (*p-value* < 0.01), and in particular results on female pelvis were higher than rectal cancer (AUC = 0.96 vs 0.72, respectively), but both groups showed a heterogeneity higher than 75%. Focusing on the rectal area, all papers applied both spatial and intensity methods, except Song et al. [69] which performed also features normalization, yielding more robust performances on a larger population. Regarding the female pelvis, we observed that the two included studies applied intensity normalization using the *z-score* method, in combination with either spatial or feature normalization. In particular, the latter approach allowed reaching higher results (AUC = 0.99 vs AUC = 0.92). However, since there were only two papers in this group, we cannot strongly assume that the combination of *z-score* and features normalization is more suitable for this organ.

Considering all the above results, it is not possible to extract clear indications on the type of pre-processing for reducing abdominal MRI variability, mainly because we found a multiplicity of algorithms and pipelines applied. Moreover, no definite correlation between normalization and performance emerges from the meta-analyses, since most of the considered subgroups were very small. Both aspects could be partially associated to the quite recent interest in multi-center studies concerning abdominal MRI, increased only from 2020.

The prostate was the only organ showing a low heterogeneity with a sufficiently high number of included papers: in this case, the most used methods for the characterization aim are *resampling* for spatial and *min-max* for features normalizations. Conversely, intensity normalization was preferably applied using *custom* algorithms, tailored by each research group considering the characteristics of the organ, i.e., its heterogeneity and the presence of small lesion difficult to be detected even by experts, and clinical task. For these reasons, we strongly recommend to carefully analyze the available images and aim.

Regarding both the rectal are and female pelvis, we noticed that *resampling and resize* have become a standard step since 2021. Moreover, the combination with *z-score* intensity normalization seems to lead to higher results for characterization and therapy response aims.

In recent years, several efforts have been made toward the implementation of new approaches based on DL algorithms, i.e., transfer learning, data synthesis, thanks to their capability to learn from a given datasets [51], [82], [83]. Despite recent success, DL is not always a feasible approach for every clinical aim, since it requires a large amount of well annotated data. This is not a straightforward task for different reasons, including the lack of data for rare diseases, and lack of clinical check on the reference standard of most publicly available datasets.

This systematic review highlighted the shortage of evidences and guidelines on normalization approaches for abdominal MRI, differently from the agreed pipelines related to the brain [14], [97]. Up to now, an initial agreement was obtained by introducing Image Biomarker Standardization Initiative (IBSI), a protocol which works towards standardizing the extraction of radiomics features but does not suggest any pre-processing pipelines [98].

Several insights emerge from our analyses. First, it could be useful to evaluate how the different normalizations really affect the model performances. In this review, it was not possible to carry out this evaluation per each study since most of them did not present the results without applying the normalization. Then, despite being able to identify the most commonly used normalization approaches, we could not provide precise explanations of the reason behind, since the majority of the collected papers did not justify the choice or provided evidences of pros and cons of different methodologies. Finally, the number of studies included in the two meta-analyses is lower than the reviewed articles, since not all of them evaluated the performances using the same metric and externally validated their models.

Our preliminary findings should be further validated using a larger amount of paper. This could be achieved by including other normalization methods applied on different AI-based system development steps, such as feature selection and dimensionality reduction, [99], and feature filtering [100].

## IV. Conclusion

Recently the need to define a suitable and useful normalization method for the reduction of multi-center MRI database variability for all abdominal organs has increased. Thanks to the findings obtained by the systematic review and meta-analyses carried out on subgroups of papers, we observed that there are some commonly used approaches, but not clear guidelines on different methodologies. Therefore, the definition of an abdominal pre-processing pipeline is still ongoing research, and it is of crucial importance to keep working on defining a proper methodology to reduce the multi-center database variability. In conclusion, we suggest carefully selecting the proper normalization approach considering the MRI database provided, the clinical aim, and the radiomics model.
